# Advanced 3D Photogrammetric Surface Reconstruction of Extensive Objects by UAV Camera Image Acquisition

**DOI:** 10.3390/s18092815

**Published:** 2018-08-26

**Authors:** Michele Calì, Rita Ambu

**Affiliations:** 1 Department of Electric, Electronics and Computer Engineering, University of Catania, V.le A. Doria, 6, 95125 Catania, Italy; 2 Department of Mechanical, Chemical and Materials Engineering, University of Cagliari, via Marengo 2, 09123 Cagliari, Italy; ambu@unica.it

**Keywords:** accuracy, Ground Sampling Distance, Structure-from-Motion algorithms, acquisition grid optimization, Digital Surfaces Models

## Abstract

This paper proposes a replicable methodology to enhance the accuracy of the photogrammetric reconstruction of large-scale objects based on the optimization of the procedures for Unmanned Aerial Vehicle (UAV) camera image acquisition. The relationships between the acquisition grid shapes, the acquisition grid geometric parameters (pitches, image rates, camera framing, flight heights), and the 3D photogrammetric surface reconstruction accuracy were studied. Ground Sampling Distance (*GSD*), the necessary number of photos to assure the desired overlapping, and the surface reconstruction accuracy were related to grid shapes, image rate, and camera framing at different flight heights. The established relationships allow to choose the best combination of grid shapes and acquisition grid geometric parameters to obtain the desired accuracy for the required *GSD*. This outcome was assessed by means of a case study related to the ancient arched brick *Bridge of the Saracens* in Adrano (Sicily, Italy). The reconstruction of the three-dimensional surfaces of this structure, obtained by the efficient Structure-From-Motion (SfM) algorithms of the commercial software Pix4Mapper, supported the study by validating it with experimental data. A comparison between the surface reconstruction with different acquisition grids at different flight heights and the measurements obtained with a 3D terrestrial laser and total station-theodolites allowed to evaluate the accuracy in terms of Euclidean distances.

## 1. Introduction

Automated photogrammetry using UAV image acquisition for digital surface reconstruction has become more widespread in recent years. This can be attributed to the enhanced performance of UAV [1,2,3] and to the development of different computer vision algorithms [4] and computational techniques, which have greatly speeded up the processing time and the quality of the reconstruction [5,6,7].

These techniques have been used for different purposes, including shape detection [8,9] and 3D surface reconstruction of large-scale elements, where a high number of photos is necessary, such as natural environments and geographical configurations [10,11,12], buildings and urban textures [13,14,15], archaeological sites [16,17], and industrial installations [18,19]. In many of these applications, there is an urgent need for the reconstruction of 3D structures from the 2D images collected from a UAV camera quickly and with a great accuracy.

When a UAV is used simply as a platform to acquire images along with a pre-programmed grid and the GPS-enabled trajectory is at a predetermined frame rate [20], it is likely that the acquisition of more images and/or their integration with other images may be necessary to obtain the required accuracy in the three-dimensional reconstruction [21]. When both the dimensions of the object being reconstructed and the accuracy increase, the computational time of the algorithms also increases significantly, thus limiting them to high-speed reconstruction applications.

Researchers have proposed the use of improved algorithms for different situations based on early SfM algorithms [22]. A variety of SfM strategies have emerged, including incremental [23,24], hierarchical [25], and global [26,27,28] approaches. Actually, the procedures developed in the computer vision community have focused more on the speed of the implemented procedures and on the success in image orientation.

To the knowledge of the authors, no scientific work up to now has presented systematic data concerning the improvement of results in a photogrammetric reconstruction against different acquisition grid shapes and acquisition grid geometric parameters (pitches, image rates, camera framing, flight heights).

The goal of the research was to determine the relationship between acquisition grid shapes, acquisition grid geometric parameters, and the accuracy obtained in the 3D reconstruction of large objects’ surfaces. The parameters investigated were therefore the grid geometric parameters: pitches, image rates, camera framing, and flight heights. The simplest three types of grids shapes (rectangular, elliptical, and cylindrical) were intended to be compared each other by studying, for each of them, the influence of pitches, image rates, camera framing, and flight heights on the accuracy of the surfaces’ reconstruction. The study highlighted how much Ground Sampling Distance (*GSD*), the necessary number of photos to assure the desired overlapping, and the surface reconstruction accuracy were related to grid shapes, image rate, and camera framing at different flight heights (15 m, 20 m, 30 m, 40 m, and 50 m).

The case study of the *Bridge of the Saracens* in Adrano (Sicily), a valuable example of Roman architecture characterized by an elongated longitudinal shape, geometric singularities, multiple and variously inclined features, and different lighting levels, offered relevant results referring to the examined relationships to support the methodology by validating it with experimental data.

Images, obtained by a CMOS 12 MP sensor, were used in Pix4Dmapper version *3* commercial software to generate dense point clouds. Function Based Method (FBM) [29] and Area Based Matching (ABM) [30,31] algorithms were employed to evaluate the degree of overlapping in the image acquired. Some cutting edge matching techniques with binary descriptors were used to quickly and accurately match keypoints.

The quality of Digital Surfaces Models (DSM) obtained from UAV image acquisitions was evaluated by comparing the photogrammetric reconstructions to the data acquired by the 3D laser scanner and total station-theodolites.

The paper presents the following structure divided into five sections, excluding the introduction. Section 1 is devoted to a description of the materials and methods used. In the Section 2, the process of image acquisition and surface reconstruction models are illustrated. In the Section 3, by means of a statistical synthesis of the surface reconstruction data, the relationship between acquisition grid shapes, acquisition grid parameters, and accuracy in 3D reconstruction is analyzed and discussed. Final considerations and conclusions are drawn in Section 4 and Section 5, respectively.

## 2. Materials and Methods

**Figure 1:**
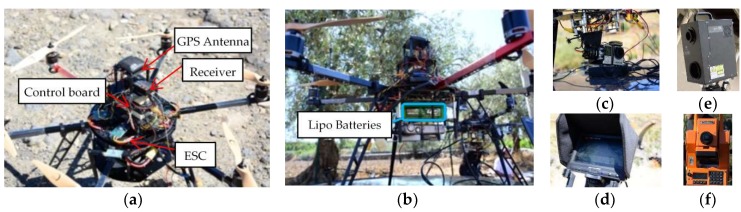
(**a**) UAV platform; (**b**) batteries and camera; (**c**) gimbal and camera; (**d**) LCD screen on the radio control; (**e**) laser scanner Konica Minolta 9v-I; (**f**) Geodimeter 480 total station-theodolites.

The fieldwork involved the following steps: acquisition, reconstruction, and analysis sessions. Initially, the aerial photo shooting was performed with different grid geometric parameters and configurations. For the aerial photogrammetric acquisition, an amateur UAV Hexacopter with Lipo 4S cells (4000 mha, 14.4 V, 576 Wh—over 20 min autonomy) was used (Figure 1a,b)

The control board was an Ardupilot APM 2.6 with Arducopter 3.1.5 flying software and a PC Mission Planner ground station. The board was equipped with a gyroscope, an accelerometer, a magnetometer, a barometer which supplied the processor with 3D data concerning position and acceleration, and an electric speed controller (ESC) HobbyKing F30 (HK, Hong Kong, China) for the brushless motor.

An action camera GoPro Hero 4 (Woodman Labs, San Francisco, CA, USA) Black Edition (Figure 1c) was positioned beneath the drone on a ‘Gimbal’ support. This support allowed the camera to rotate along three axes (pitch, roll, yaw) controlled by a digital board and manoeuvred by brushless motors which could dampen any drone shift keeping the camera motionless. The camera had a CMOS 12 MP 1/2.9″ solid-state sensor which could even sense electromagnetic data, so it was also possible to check the radiometric data in the image pixels. A video transmitter provided real time shots on a 7″ LCD monitor incorporated into the radio control unit (Figure 1d). The UAV platform and camera characteristics are summarized in Table 1 and Table 2, respectively.

**Figure 2:**
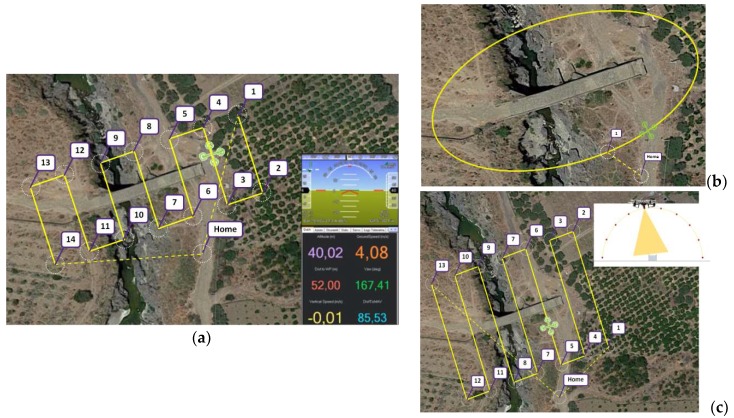
Waypoint acquisition grids: (**a**) Rectangular at $h_{v}$ = 40 m; (**b**) Elliptical at $h_{v}$ = 30 m; (**c**) Cylindrical $h_{v}$ = 40 m.

GPS LEA6H (uBlox, Thalwil, Switzerland) with Ground Station PC Mission Planner software (version 1) converted a discrete number of points into geo-referenced coordinates (GRC) by generating waypoint acquisition grids. In this way, it was possible to define different configurations of 3D grids. From each GRC, an image was acquired [32]. Figure 2a shows an example of a rectangular waypoint acquisition grid at a flight height of 40 m. The numbers in the figure indicate the points in which the drone started, finished, and changed its trajectory. Figure 2b shows, instead, an elliptical waypoint acquisition grid at a flight height ($h_{v}$) of 30 m.

The commercial software Pix4Dmapper was used to create a polygonal mesh of the surface from image collections captured by UAVs. The method employed for mosaicking images was Bundle Block Adjustment (BBA) with SfM algorithms [33]. Each image has six exterior parameters: the camera’s position along with its roll, pitch, and yaw angles, which mapped each scene point (*x*, *y*, *z*) to the corresponding image point (*x*’, *y*’). The parameters of these equations were as follows: (*x*_c_, *y*_c_, *z*_c_) was the camera’s position and (*m*_ij_) was the rotation matrix 3 × 3 defined by the roll, pitch, and yaw angles of the camera. There were more fixed parameters: (*x*_p_, *y*_p_) was the main point, whereas *f*_x_ and *f*_y_ were focal length ratios. The focal points and focal lengths, along with the distortion and radius of the radial lens, were determined by a calibration procedure before each acquisition flight.

By means of the described method, 20 different polygonal meshes were generated in correspondence of 20 different waypoint acquisition grids by combining three different acquisition grid shapes (rectangular, elliptical, and cylindrical), five flight heights (15 m, 20 m, 30 m, 40 m, and 50 m), and different camera framing. The relationship between acquisition grid shapes and acquisition grid geometric parameters, as well as *GSD* and 3D reconstruction accuracy at a certain image overlap value, was determined.

For rectangular camera grid acquisitions, different camera framing orientations were used, obtaining two different image collection sets. The first set of images was acquired by vertically oriented aerial photo shooting, which will hereinafter be referred to as the “Rectangular Grid with Vertical Camera” (RGVC). The RGVC was capable of generating orthomosaic image collections. The second image collection set was acquired by a camera oriented differently for each image using a gimbal and action camera, which will hereinafter be referred to as the “Rectangular Grid with Oscillating Camera” (RGOC).

The image collection sets in elliptical and cylindrical camera grid acquisitions were made exclusively by a camera oriented differently for each image, and will hereinafter be referred to as the “Elliptical Grid” (EG) referring to the first acquisition grid, and the “Cylindrical Grid” (CG) referring to the second acquisition grid.

In the case study of the bridge surface reconstruction, the accuracy of the obtained twenty surface reconstructions was evaluated with the data acquired using a 3D laser scanning device Konica Minolta 9v-I (Konica Minolta, Ramsey, NJ, USA), precision ≤2 mm (Figure 1e), and a total station Geodimeter 480 (Geoglobex, Monza, Italy), distance accuracy ≤3 mm (Figure 1f).

## 3. Image Acquisition and Surface Reconstructions

### 3.1. Image Acquisition

**Figure 3:**
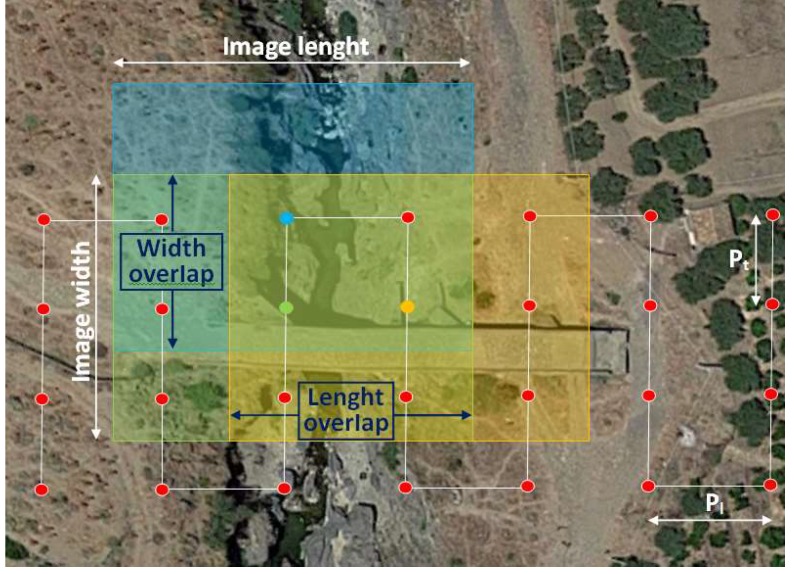
Waypoint and overlap in RGVC at $h_{v}$ = 40 m.

In the case study of the bridge surface reconstruction, the aerial image acquisition phases are illustrated as follows. Aerial image acquisitions were made with RGVC, RGOC, EG, and CG at five flight heights: 15 m, 20 m, 30 m, 40 m, and 50 m. The acquisition pitches *p* (m) in the grids forming the waypoints (Figure 3) were a function of the required Ground Sampling Distance (*GSD*) (cm/pixel) and *overlap*. They were calculated according to the following Equation (1) [34]:

(1) $$p=\left(\frac{ImW_{p}\times GSD}{100}\right)\times \left(1-overlap\right)$$

where $ImW_{p}$ was the image width [pixel]. When the acquired images had a different length and width (ImL and ImW), it was necessary to define two different pitches to have a constant value of the overlap along the two orthogonal directions in the acquisition grids (*Width Overlap* and *Length Overlap*). In the RGVC considering the longitudinal extension as the reference point and positioning the grids as shown in Figure 2a, it was possible to determine the longitudinal pitch (*p_l_*) and (orthogonal to the first) a transversal pitch (*p**_t_***), which ensured a constant overlap value of 66% (Figure 3). In Figure 3 are highlighted three areas, respectively represented with blue, orange and green colour, corresponding to different acquired images and the zone of their overlap, which defines the length and width of overlapping. The dots represent the subsequent positions of the UAV from which the three images were acquired. In the present study, in which the image dimensions in pixel ($ImL_{p}$ and $ImW_{p}$) were 4000 pixels and 3000 pixels, respectively, the following acquisition pitch *p* values were established:

(2) $$p_{l}=\left(\frac{ImL_{p}\times GSD}{100}\right)\times \left(1-overlap\right)=\;\left(\frac{4000\times GSD}{100}\right)\times 33.3$$

(3) $$p_{t}=\left(\frac{ImW_{p}\times GSD}{100}\right)\times \left(1-overlap\right)=\left(\frac{3000\times GSD}{100}\right)\times 33.3$$

where *GSD* (cm/pixel) was calculated according to the following Equation (4) as a function of $h_{v}$:

(4) $$GSD=\frac{h_{v}\times S_{w}\times 100}{F_{l}\times ImW}$$

In this equation, $S_{w}$ was the sensor width and $F_{l}$ was the focal length (See Table 2). Therefore, in RGVC, an exactly constant value of the overlap value equal to 66% along the two orthogonal directions (Table 3a) was obtained.

**Figure 4:**
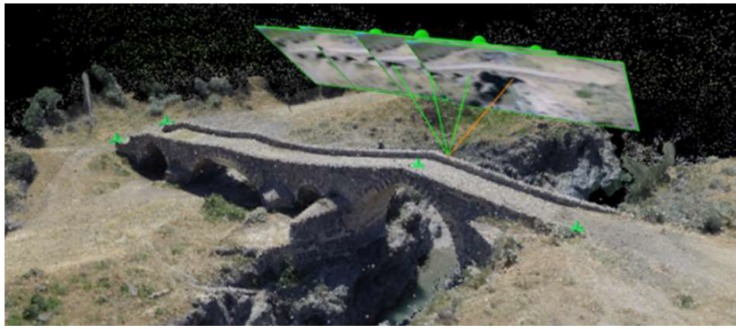
Common keypoint in three sequentially captured images in EG at $h_{v}$ = 30 m.

In RGOC, EG, and CG, the values of grid shapes and acquisition pitches *p* were established to ensure overlap values close to 66% along the two orthogonal directions. These values of overlap close to 66% in RGVC, RGOC, EG, and CG ensured that each keypoint was captured in at least three different shots (Figure 4), thus avoiding the risk of insufficient feature overlaps across images in post-flight image processing, as well as the subsequent risk of failure in 3D reconstruction. This result is also reported in other research projects [35], to ensure full coverage in aerial laser scanning.

**Figure 5:**
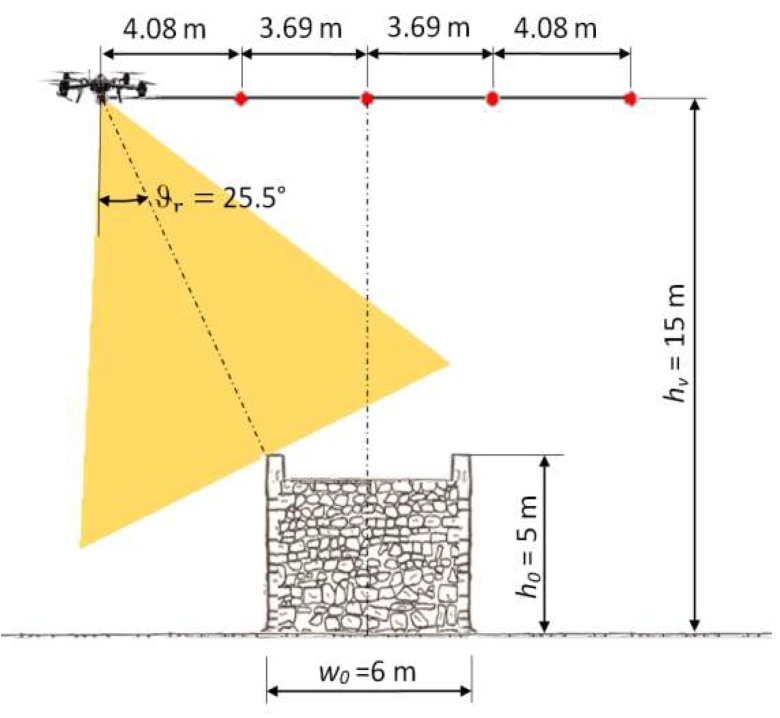
Gimbal rotation *ϑ_r_* around the horizontal axis parallel to the longitudinal direction in EG at $h_{v}$ = 15 m.

In the RGOC, the overlap value equal to 66% was imposed along the transversal direction, determining the values of transversal pitches (*p**_t_***). Using longitudinal pitches (*p_l_*) equal to RGVC, it was checked that the value obtained along the longitudinal direction was never less than 66% (Table 3b). The gimbal rotation *ϑ_r_* around the horizontal axis parallel to the longitudinal direction was synchronized with the transverse distance *x* (m) of the UAV platform in relation to the center line and with the flight height *h_v_* (m) according to Equation (5):

(5) $$\vartheta_{r}=\tan^{-1}\frac{x-w_{o}/2}{h_{v}-h_{o}}$$

where *h_o_* (m) stands for the object height and *w_o_* (m) stands for the object width (Figure 5).

**Figure 6:**
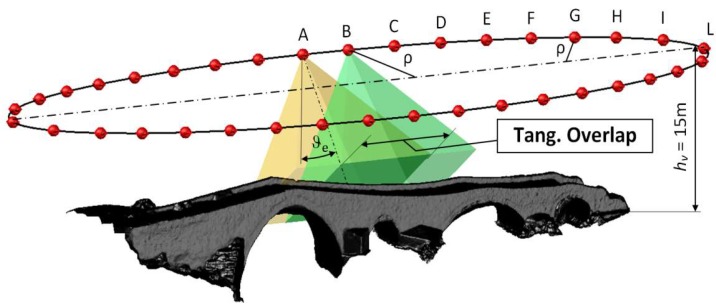
Waypoint and overlap in EG at $h_{v}$ = 15 m.

In EG (Figure 6), the overlap value equal to 66% was imposed along the tangential direction (*Tangential Overlap)*, thus obtaining the value of the tangential pitch (*p_tan_*), and it was checked that the overlap value obtained along the transversal direction (*Transversal Overlap*), orthogonal to the first, was never less than 66% (Table 3c). In EG, the gimbal rotation *ϑ_e_* around the tangential axis to the elliptic orbit was synchronized with the minimum distance *ρ* from the acquired object according to the following Equation (6):

(6) $$\vartheta_{e}=\tan^{-1}\frac{\rho }{h_{v}-h_{o}}$$

Equation (6) was also used to determine the values of the ellipses axis at the various flight heights. In particular, the axis lengths were chosen to obtain a value of the gimbal rotation *ϑ_e_* around the tangential axis close to 30° at point A and close to 40° at point L. It was seen that these values allowed us to obtain both the overlap values (tangential and transversal) close to 66% at the same time.

**Figure 7:**
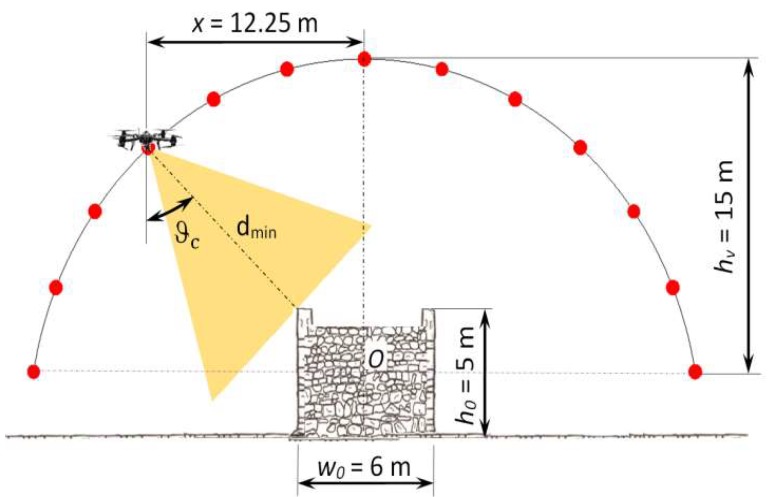
Gimbal rotation *ϑ_c_* in CG at $h_{v}$ = 15 m.

In CG, the gimbal rotation *ϑ_c_* around the horizontal axis parallel to the longitudinal axis was synchronized with the radial direction of the cylindrical trajectory according to the following Equation (7) (Figure 7):

(7) $$\vartheta_{c}=\tan^{-1}\frac{x-w_{o}/2}{h_{v}-h_{o}/2}$$

**Figure 8:**
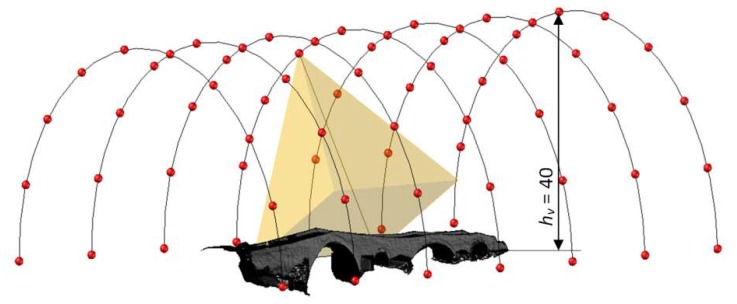
Waypoint in CG at $h_{v}$ = 40 m.

By replacing the minimum distance from the bridge (*d_min_*) in Equation (1) with the value *h_v_*, pitch values that ensured overlap values close to 66% were determined. In Figure 8, the waypoint at $h_{v}$ = 40 m is shown. Values of *GSD* overlap and pitches are shown in Table 3d.

Table 4 shows the geometric parameters (grid pitches, width, and length) in RGVC, RGOC, EG, and CG. The number of photos (n. GRC) for each acquisition set in order to obtain the overlap values close to 66% is also reported.

**Figure 9:**
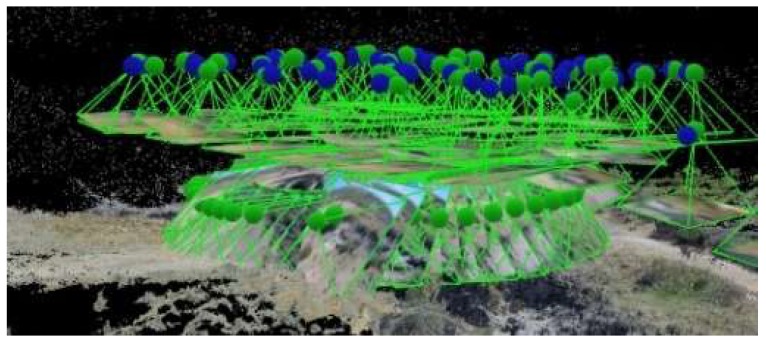
EG at $h_{v}$ = 30 m and RGVC at $h_{v}$ = 50 m.

Figure 9 shows the EG at 30 m and the RGVC at 50 m.

### 3.2. Surface Reconstruction

**Figure 10:**
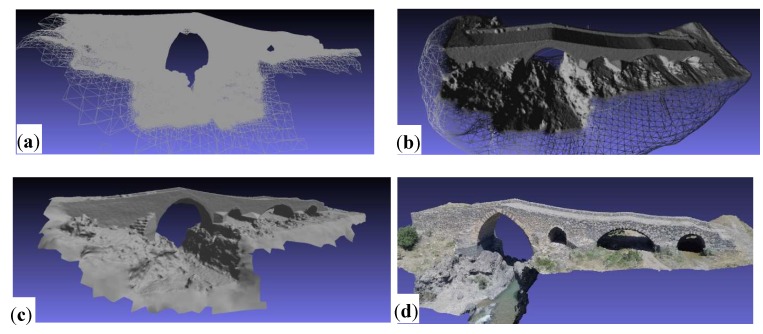
Surface reconstruction: (**a**) polygonal mesh of surfaces; (**b**) polygonal mesh visualized with shade; (**c**) nurbs surface; (**d**) mapped texture surface.

Once the image collections for the reconstruction were acquired, the system almost automatically calibrated the cameras based on the exchangeable image file (EXIF) information, also reported in [7], and the Fisheye lens camera model (Pix4D), found in the images, and the GCPs aligned them into the 3D space and produced a complete and single mesh using SfM algorithms. The reconstruction of the surface is based on the following steps:

1. Algorithm searches for matching points by analyzing all the acquired images. Matching techniques with binary descriptors, together with FBM and ABM, are employed to identify features irrespective of their position, scale, and rotation. Studies on the performance of such feature descriptors are given in [36].
2. Matching points, as well as approximate values of the image position and orientation provided by Ardupilot APM 2.6, are used in BBA (Bundle Block Adjustment) [37,38] to reconstruct the exact position and orientation of the camera for every acquired image.
3. Based on this reconstruction, the matching points are verified and their 3D coordinates are calculated. The geo-reference system used is uBlox LEA6H with Groundstation PC Mission Planner software (version 1), based on GPS measurements from the UAV Ardupilot APM 2.6 during the flight.
4. Those 3D points are interpolated to form a triangulated irregular network in order to obtain a polygonal mesh of surfaces (Figure 10a,b) [39,40]. At this stage, a dense 3D model can increase the spatial resolution of the triangle structure [41].
5. The polygonal mesh of surfaces is used to project every image pixel, obtaining a mapped texture surface (Figure 10d), and to calculate the georeferenced orthomosaic [42].

In detail, from the 3D feature points calculated by the SfM, the mesh data were obtained by using Delaunay triangulation. Then, the mesh was used as an outline of the object, which was projected onto the plane of the images to get the estimated depth maps. These maps were optimized and corrected using the pixel matching algorithm based on the patch. Finally, dense point cloud data were obtained by fusing these depth maps. From the detected accurate cloud points, a 3D polygonal mesh was obtained (point 4 of previous reconstruction steps). The polygonal mesh obtained can be easily transformed, through open source algorithms, into a nurbs surface (Figure 10c) for different applications. Figure 10 shows some steps of the described procedure in the case study of the bridge surface reconstruction (CG at $h_{v}$ = 15 m).

**Figure 11:**
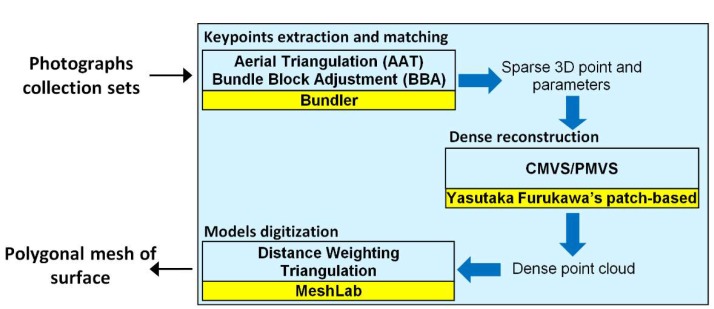
Flowchart of the main steps of the surface reconstruction algorithm.

The flowchart relative to the reconstruction algorithm is summarized in Figure 11. Within the blocks highlighted in yellow, the equivalent open source algorithm is shown.

In the Reference [43], it is possible to find the necessary information to also implement the reconstruction algorithms used in open source software, for obtaining results similar to those of the commercial software. In the same paper, the matching features algorithm written in pseudocode can be found.

## 4. Reconstructions Accuracy Evaluation

**Figure 12:**
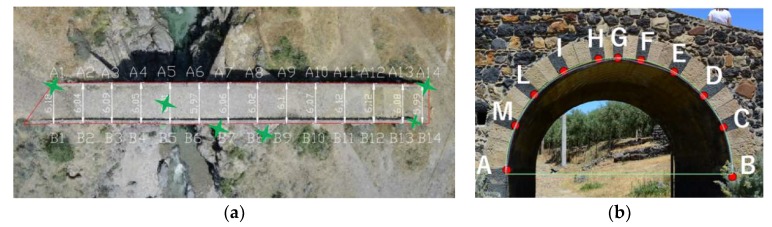
(**a**) 29 keypoints on the pounding upper surface; (**b**) 11 keypoints on the south side of the north-east arch.

The accuracy evaluation of the 20 reconstructions in the case study of the bridge was performed by measuring two reference shapes: the pounding upper surface (*pus*) and the south side of the north-east surveyed arch (*arc*) of the bridge. Using the terrestrial laser scanner and the total station-theodolites, the coordinates of 29 keypoints on the pounding upper surface (Figure 12a) and the coordinates of 11 keypoints on the south side of the north-east surveyed arch (Figure 12b) were acquired.

The choice of these two shapes, being on two mutually orthogonal positions, allowed the right accuracy evaluation of the reconstructions entirely along the three dimensions. Such shapes had, indeed, a favorable spatial distribution, which allowed an accurate validation of the length, width, and height and the reconstructed profile evaluation of the arch. Six of these points (outlined with green markers in Figure 10a) were those used as GCPs.

The partial scans were acquired by positioning the scanner’s sensor plane parallel to the upper surface. The comparison was carried out using Meshlab [44] and CloudCompare [45,46,47,48] open source software. Moreover, Meshlab was used to align the partial scans with the 3D model produced by Pix4Dmapper. Instead, the CloudCompare software was employed to estimate the surface deviation between the mesh obtained with Pix4Dmapper and the point cloud obtained with 3D laser scanning.

The ICP algorithm, implemented in Meshlab, was used to align each partial view obtained with the 3D laser scanning with the Pix4Dmapper triangular mesh model. In order to compare the two data types, the cloud-to-mesh distance function offered by CloudCompare was selected as it was considered as more robust to local noise. The cloud-to-mesh distance function computed the distances between each vertex of the point cloud to the nearest triangle of the mesh surface. The distance between the two was calculated as follows. In cases where the orthogonal projection of the vertex laid inside the surface defined by a triangle, then the distance between the vertex and its point of intersection on the surface was calculated. Accuracy (*acc*) was measured by evaluating these distances between the obtained points and the homologous points in the 3D reconstructions. For a precise accuracy evaluation, for both the two shapes, the accuracy was evaluated by introducing the standard deviation (*σ*) of such differences for a typical length of the shape (Equations (8) and (9)). With reference to the pounding upper surface (*pus*), the typical distance considered was the mean value ${\bar{AB}}_{mean}$ of the 14 distances $\bar{A_{x}B_{x}}$ (*x* = 1, 2, …14), and for the arch (*arc*), the observed typical distance was the mean value *R_mean_* of the 11 rays *R_y_* (*y* = A, B, …M).

(8) $$acc_{pus}=\frac{14}{\sigma_{\bar{AB}}\times \sum_{x=1}^{14}\left({\bar{A_{x}B_{x}}}_{measured}-{\bar{A_{x}B_{x}}}_{recontruction}\right)}=\frac{1}{\sigma_{\bar{AB}}\times {\bar{AB}}_{mean}}\;\mathrm{for}\;x=1,\;2,\;\ldots 14$$

(9) $$acc_{arc}=\frac{11}{\sigma_{R}\times \sum_{y=A}^{M}\left(R_{y\;measured}-R_{y\;reconstruction}\right)}=\frac{1}{\sigma_{\bar{AB}}\times R_{mean}}\;\;\;\;\mathrm{for}\;x=A,\;B,\;\ldots M$$

**Figure 13:**
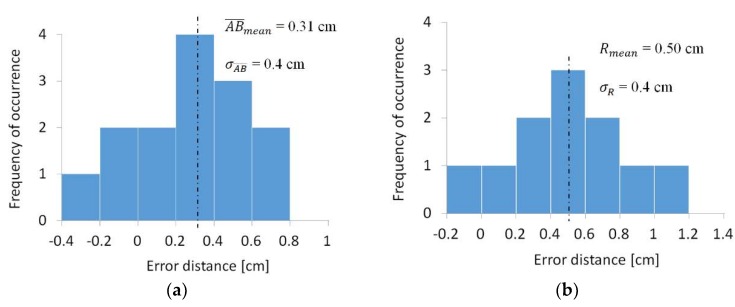
Error in mean distances (cm) and its standard deviation distances (cm) distribution graphs in CG at 15 m: (**a**) pounding upper surface; (**b**) south side of the north-east surveyed arch.

Figure 13 shows the mean distance (cm) and standard deviation distances ($\sigma_{\bar{AB}}$; $\sigma_{R}$) calculated in cm in the case of surface reconstruction with CG at $h_{v}$ = 15 m.

In Table 5, the values of the mean error distance (${\bar{AB}}_{mean}$; $R_{mean}$) and standard deviation distances ($\sigma_{\bar{AB}}$; $\sigma_{R}$) expressed in cm in the 20 reconstructions are shown. These values enabled us to measure the accuracy through each of the 20 reconstructions which were taken into consideration in the present work.

In particular, the mean error of distances (${\bar{AB}}_{mean}$; $R_{mean}$) indicated the mean value of the accuracy of every acquisition method, and the standard deviation error of distances ($\sigma_{\bar{AB}}$; $\sigma_{R}$) indicated the extent of the error distribution. The most accurate reconstructions were those characterized by smaller values of mean error for distance and smaller values of standard deviation error. Table 5 shows values of $acc_{pus}$ (cm) and $acc_{arc}$ (cm) evaluated in cm with Equations (8) and (9), respectively.

When considering a comparison of the 20 different types of surface reconstruction related to the number of photos used (n. GRC) and the mean value of *GSD* offered by each of them, the following parameters were calculated ξ (cm^2^) by multiplying the accuracy *acc* by *GSD* and the number of photos (n. GRC):

(10) $$\xi_{pus\;}=acc_{pus}\times GSD\times n.\mathrm{GRC}$$

(11) $$\;\xi_{arc\;}=acc_{arc}\times GSD\times n.\mathrm{GRC}$$

The inferior values of these parameters allowed us to improve the accuracy and the speed of reconstruction at the same time. In Table 6, the values of the products ξ (cm^2^) for the 20 different types of surface reconstruction are shown.

The parameters ξ enabled us to qualify every method which was analyzed in this study, regardless of the value of *GSD*, which did not include the acquisition orientation, and the position in relation to the part in acquisition.

## 5. Data Comparison and Discussions

**Figure 14:**
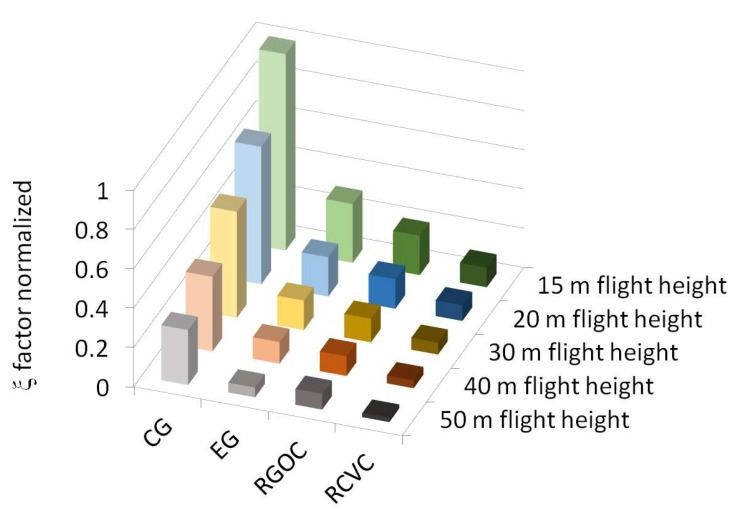
ξ factor normalized.

The comparative analysis of the data obtained from the twenty reconstructions highlighted some interesting points of discussion and provided useful information for the photogrammetric surface reconstruction of large-scale objects. In all the reconstructions which were studied, the accuracy resulted in Gaussian-like distributions. The accuracy was always proportional to *GSD*, but the number of the images which needed to be acquired to obtain the desired accuracy varied according to the grid shapes and the acquisition parameters utilized. Normalizing at one the sum of the two factors ($\xi_{pus}$ + $\xi_{arc}=\xi$), it appeared clear (Figure 14) that the acquisition with CG enabled us to obtain a quality of the reconstruction highly superior at all the flight heights. In Figure 14 the values of ξ factor as normalized for the grid shapes at 15 m flight height were showed in green colors. The values of ξ factor normalized for the grid shapes at 20 m flight height were showed in blu colors. The values of ξ factor normalized for the grid shapes at 30 m flight height were showed in brown colors. The values of ξ factor normalized in the grid shapes at 40 m flight height were showed in red colors and lastly the values of ξ factor normalized in the grid shapes at 50 m flight height were showed in grey colors. On average, the CG grid shape improved the accuracy by more than a factor of six/seven.

The image overlaps were proportional to flight height and inversely proportional to grid pitch studied. The synergic effects of grid shapes, grid pitch, and camera framing, instead, could not always be predicted and their right combination might provide an advanced accuracy in photogrammetric surface reconstruction.

The equations from (1) to (7) permitted us to correlate *GSD*, flight altitudes, and overlap with one another. In particular, it was possible to use such equations to locate the flight heights, which assured the desired values of *GSD* and overlap.

The acquisition with a single RGVC did not often suffice to reach the desired accuracy. In fact, although it produced acceptable values of *GSD*, it lacked greatly in the acquisition of surfaces orthogonal to the shot direction, as well as the side wall of the bridge.

Moving on from the acquisition with RGVC to the acquisition with RGVO, it was possible to keep the transversal overlap steady (66%) when increasing the transversal pitch. This occurred according to Equation (1), at the expense of *GSD*. Moreover, the rotation of the camera of the angle *ϑ**_r_*** according to Equation (5), involved an increase of the longitudinal overlap.

Similar to image overlap, the surface deviation values and the distances between feature points appeared to be inversely proportional to flight height and highly dependent on grid acquisition type. Overlap in the acquisitions with constant grid pitch seemed to be extremely variable, especially in the elliptical grid. In the acquisition around the bridge with elliptical grids, some efficiency in terms of number of images against the surface area being covered was lost.

The conducted analysis allowed us to check that all reconstructions contained errors proportional to flight height, but this was true, especially for RGVC. This acquisition, being one of the most used types, provided the worst results both in terms of accuracy (*acc*) and parameter ξ.

The acquisition with CG allowed us to get the best results. This kind of grid, thanks to the modern technologies and to a good GPS system, was easily implementable with a high accuracy in a system of acquisition by UAV.

## 6. Conclusions

A replicable and generalizable methodology was illustrated in order to improve the quality of the 3D digital surface reconstruction of large-scale objects by the photogrammetric technique. Using commercial software (Pix4Dmapper) based on the Structure-from-Motion algorithms, the existing relationships between the grid shapes, the acquisition grid parameters, the image overlap, and the accuracy of reconstruction were evaluated and discussed. The proposed relationships enabled us to obtain the appropriate selection of flight heights, acquisition grid shape, and camera framing in correspondence to a pre-established overlap value and required *GSD*.

The experiments conducted on the reconstruction of the Bridge of the Saracens in Adrano (Sicily) illustrated the effectiveness of the developed methodology, which enhanced the 3D reconstruction of a highly complex architectural structure with the desired accuracy. The errors of surface reconstruction were evaluated statistically using measures with a 3D laser scanner and total station-theodolites measurements.

The experimental results indicated that in large-scale objects characterized by an elongated longitudinal shape, geometric singularities, and multiple and variously inclined features, the proposed method could improve the accuracy, increasing the speed of reconstruction at the same time by more than a factor of six/seven. The authors believe that it might be interesting to apply the study to more complex acquisition grid shapes (i.e., a zig-zag grid).

## Figures and Tables

**Table 1:** UAV Platform.

Technical Specifications	Value/Typology
Frame	Hexacopter
Engine	T-Motors 2216 (RoHS, Hong Kong, China)
Engine size (mm)	Φ27.8 × 34
Engine Weight (g)	75
Idle current@10v (A)	0.04
Batteries	Lipo 4S—4000 mha
Max Power (Wh)	576
Rotors	Nylon10 × 5 pitch
GPS	uBlox LEA6H
Flying software	Arducopter 3.1.5
UAV Weight (g)	1120

**Table 2:** GoPro Hero 4 Black Edition.

Parameter	Value
Sensor	CMOS 12 MP 1/2.9″
Focal length (*F_l_*) (mm)	15.5
Sensor width (*S_W_*) (mm)	16.8
Sensor length (*S_l_*) (mm)	22.4
ISO sensitivity	80–6400
Lens range	f/2.0–f/5.9
Burst shooting (fps)	2.3
Weight (g)	198

**Table:** (a)

*h_v_* (m)	*GSD* (cm/pixel)	*ImW* (m)	*ImL* (m)	*W. Over.* (%)	*L. Over.* (%)	*p_t_* (m)	*p_l_* (m)
15	0.36	10.8	14.5	66.0%	66.0%	3.7	4.9
20	0.54	16.3	21.7	66.0%	66.0%	5.5	7.4
30	0.90	27.1	36.1	66.0%	66.0%	9.2	12.3
40	1.26	37.9	50.6	66.0%	66.0%	12.9	17.2
50	1.63	48.8	65.0	66.0%	66.0%	16.6	22.1

**Table:** (b)

*h_v_* (m)	$\vartheta_{r}\left({}^{\circ}\right)$	*GSD* (cm/pixel)	*ImW* (m)	*ImL* (m)	*W. Over.* (%)	*L. Over.* (%)	*p_t_* (m)	*p_l_* (m)
15	0.0	0.36	10.8	14.5	66.0%	66.0%	3.7	4.9
	4.0	0.36	10.9	14.5	66.0%	66.1%	3.7	4.9
	25.5	0.40	12.0	16.0	66.0%	69.3%	4.1	4.9
20	0.0	0.54	16.3	21.7	66.0%	66.0%	5.6	7.5
	9.9	0.55	16.5	22.0	66.0%	66.0%	5.6	7.5
	31.2	0.63	19.0	25.3	66.0%	70.5%	6.5	7.5
30	4.0	0.93	28.0	37.3	66.0%	66.0%	9.5	12.3
	25.6	1.00	30.0	40.1	66.0%	68.3%	10.2	12.3
40	6.1	1.32	39.6	52.8	66.0%	66.0%	13.5	17.2
	35.6	1.43	42.8	57.1	66.0%	68.5%	14.6	17.2
50	7.2	1.71	51.2	68.3	66.0%	66.0%	17.4	22.1
	28.7	1.85	55.6	74.1	66.0%	68.7%	18.9	22.1

**Table:** (c)

*h_v_* (m)	$\vartheta_{e}\left({}^{\circ}\right)$	*GSD* (cm/pixel)	*ImW* (m)	*ImL* (m)	*Tan. Over.* (%)	*Trans. Over.* (%)	*p_tan_* (m)
15	23.2	0.41	12.3	16.4	66.0%	66.1%	4.2
	35.0	40.76	15.3	20.4	66.0%	78.3%	5.2
20	27.7	0.67	20.1	26.8	66.0%	66.0%	6.8
	38.7	0.77	23.0	30.7	66.0%	80.3%	7.8
30	33.6	1.15	34.4	45.9	66.0%	66.1%	11.7
	41.3	1.28	38.3	51.1	66.0%	80.1%	13.0
40	38.7	1.69	53.6	71.5	66.0%	66.0%	17.2
	42.4	1.79	50.7	67.6	66.0%	79.8%	18.2
50	38.7	2.15	64.5	86.1	66.0%	66.0%	21.9
	43.0	2.30	69.0	92.0	66.0%	80.6%	23.5

**Table:** (d)

*h_v_* (m)	$\vartheta_{e}\left({}^{\circ}\right)$	*GSD* (cm/pixel)	*ImW* (m)	*ImL* (m)	*W. Over.* (%)	*L. Over.* (%)	*p_t_* (m)	*p_l_* (m)
15	0.0	0.36	10.8	14.5	66.0%	66.0%	3.7	4.9
	90.0	0.43	13.0	17.3	66.0%	71.7%	4.4	4.9
20	0.0	0.54	16.3	21.7	66.0%	66.0%	5.5	7.4
	90.0	0.61	18.4	24.6	66.0%	70.0%	6.3	7.4
30	0.0	0.90	27.1	36.1	66.0%	66.0%	9.2	12.3
	90.0	0.98	29.3	39.0	66.0%	68.5%	10.0	12.3
40	0.0	1.26	37.9	50.6	66.0%	66.0%	12.9	17.2
	90.0	1.34	40.1	53.5	66.0%	67.8%	13.6	17.2
50	0.0	1.63	48.8	65.0	66.0%	66.0%	16.6	22.1
	90.0	1.70	50.9	67.9	66.0%	67.4%	17.3	22.1

**Table 4:** Geometric characteristics of: RGVC, RGOC, EG, and CG.

**RGVC—Rectangular Grid with Vertical Camera**						**RGOC—Rectangular Grid with Oscillating Camera**					
***h_v_* (m)**	***p_t_* (m)**	***p_l_* (m)**	**Width (m)**	**Length (m)**	**n. GRC**	***h_v_* (m)**	***p_t_* (m)**	***p_l_* (m)**	**Width (m)**	**Length (m)**	**n. GRC**
15	3.7	4.9	14.8	73.5	80	15	3.7; 4.1	4.9	15.5	73.5	80
20	5.5	7.4	22	81	60	20	5.6; 6.5	7.4	24.1	81	60
30	9.2	12.3	27.6	86	32	30	9.5; 10	12.3	41.7	86	40
40	12.9	17.2	38.7	103.2	28	40	13.5; 14.6	17.2	59.4	103.2	35
50	16.6	22.1	49.8	110.5	24	50	17.4; 18.9	22.1	77.1	110.5	30
**EG—Elliptical Grid**						**CG—Cylindrical Grid**					
***h_v_* (m)**	***p_tan_* (m)**		**Width (m)**	**Length (m)**	**n. GRC**	***h_v_* (m)**	***p_t_* (m)**	***p_l_* (m)**	**Width (m)**	**Length (m)**	**n. GRC**
15	4.2; 5.2		26	80	36	15	3.7; 4.4	4.9	30	73.5	208
20	6.8; 7.8		36	90	28	20	5.5; 6.3	7.4	40	81	144
30	11.7; 13		56	110	22	30	9.2; 10	12.3	60	86	88
40	17.2; 18.2		76	130	20	40	12.9; 13.6	17.2	80	103.2	70

**Table 5:** Error in mean distances and its standard deviations in cm in the 20 surface reconstructions.

Acq. Grid Tipology	${\bar{AB}}_{mean}$	$\sigma_{\bar{AB}}$	$acc_{pus}$	$R_{mean}$	$\sigma_{R}$	$acc_{arc}$	Acq. Grid Tipology	${\bar{AB}}_{mean}$	$\sigma_{\bar{AB}}$	$acc_{pus}$	$R_{mean}$	$\sigma_{R}$	$acc_{arc}$
RGVC 15 m	0.65	1.8	0.85	1.08	2.9	0.35	EG 15 m	0.45	1.1	2.02	0.70	1.7	0.84
RGVC 20 m	1.07	1.9	0.49	1.69	2.9	0.20	EG 20 m	0.78	1.2	1.07	1.26	1.7	0.47
RGVC 30 m	1.71	1.9	0.31	2.70	3.0	0.12	EG 30 m	1.28	1.2	0.65	2.03	1.8	0.27
RGVC 40 m	2.73	2.0	0.18	6.17	3.0	0.05	EG 40 m	1.87	1.3	0.41	4.76	1.8	0.12
RGVC 50 m	3.36	2.1	0.14	8.38	3.1	0.04	EG 50 m	2.50	1.3	0.31	6.43	1.8	0.09
RGOC 15 m	0.54	1.2	1.54	0.6	1.9	0.88	CG 15 m	0.31	0.40	4.67	0.5	0..0	2.30
RGOC 20 m	0.93	1.3	0.83	1.03	2.0	0.50	CG 20 m	0.52	0.58	2.77	0.84	1.1	1.09
RGOC 30 m	1.48	1.4	0.48	1.58	2.1	0.30	CG 30 m	0.82	0.74	1.53	1.30	1.1	0.70
RGOC 40 m	2.14	1.4	0.33	2.98	2.2	0.15	CG 40 m	1.15	0.80	1.09	2.95	1.1	0.31
RGOC 50 m	2.60	1.5	0.26	3.96	2.2	0.11	CG 50 m	1.54	0.80	0.81	3.97	1.2	0.21

**Table 6:** ξ factors for the 20 surface reconstructions.

Acq. Grid Tipology	$\xi_{pus}\;({\mathrm{cm}}^{2})$	$\xi_{arc}\;({\mathrm{cm}}^{2})$	Acq. Grid Tipology	$\xi_{pus}\;({\mathrm{cm}}^{2})$	$\xi_{arc}\;({\mathrm{cm}}^{2})$
RGVC 15 m	24.7	4.2	EG 15 m	33.5	13.9
RGVC 20 m	15.9	3.6	EG 20 m	21.5	9.4
RGVC 30 m	8.9	2.6	EG 30 m	17.4	7.3
RGVC 40 m	6.5	1.9	EG 40 m	14.3	4.1
RGVC 50 m	5.5	1.5	EG 50 m	12.3	3.5
RGOC 15 m	47.1	26.7	CG 15 m	369.0	165.3
RGOC 20 m	29.3	17.7	CG 20 m	230.2	90.5
RGOC 30 m	18.7	11.5	CG 30 m	126.5	57.8
RGOC 40 m	16.0	7.3	CG 40 m	98.8	28.1
RGOC 50 m	13.7	6.1	CG 50 m	81.0	21.0
